# OA13.02. Utilizing mind-body practices in public schools: teaching self-regulation skills and fostering resilience in our next generation

**DOI:** 10.1186/1472-6882-12-S1-O50

**Published:** 2012-06-12

**Authors:** M Sprengel, M Fritts

**Affiliations:** 1 Samueli Institute, Alexandria, USA

## Purpose

Due to multiple and increasing stressors including large classroom sizes, increased educational requirements and decreased personal attention, children in public schools are at a greater risk than ever of developing behavioral, anxiety and stress-related disorders. These problems have contributed to poor test scores and academic performance, as well as decreased high school graduation rates. The drop-out rate among U.S. students was 9.1% in 2009 and is approaching 50% in urban high schools. To remedy these problems, public school systems needs to provide their schools with more effective tools for coping with academic and social pressures. While the physical and mental benefits of mind-body practices are well-established in adult populations, comparatively little research exists on the potential benefits of these self-management skills in children, especially in urban areas. To begin to address this knowledge gap, we reviewed the published literature on mind-body practices in children.

## Methods

This review was performed in conjunction with a W.K. Kellogg Foundation funded project in which we are developing a framework for community wellness and resilience focusing on vulnerable children in underserved communities. This effort includes high-level linkages to national health policy (via active collaboration with the U.S. Surgeon General and the National Prevention Strategy). We searched five databases (PubMed, Medline, CINHAL, PyschInfo and EBSCO) for studies that used a mind-body therapy intervention, included children (ages 2 to 18), and were written in English.

## Results

17 articles met our inclusion criteria. The mind-body interventions studied included the Relaxation Response Training, Transcendental Meditation, HeartSmarts®/HeartMath Program, Mindfulness-Based Stress Reduction (MBSR), and miscellaneous yoga, meditation and mind-body interventions.

## Conclusion

School and classroom oriented programs that incorporate mind-body practices have demonstrated positive outcomes for well-being, resilience, academic performance, test scores, individual self-perception, self-regulation of negative behaviors, anxiety, stress, Attention Deficit Disorder/Attention Deficit Hyperactivity Disorder, insomnia, anger/aggressive behaviors, and chronic pain conditions.

